# Differential effects of speed and volume on transfusion‐associated circulatory overload: A randomized study in rats

**DOI:** 10.1111/vox.13191

**Published:** 2021-08-15

**Authors:** Robert B. Klanderman, Marije Wijnberge, Joachim J. Bosboom, Joris J. T. H. Roelofs, Dirk de Korte, Robin van Bruggen, Markus W. Hollmann, Margreeth B. Vroom, Denise P. Veelo, Nicole P. Juffermans, Bart F. Geerts, Alexander P. J. Vlaar

**Affiliations:** ^1^ Department of Intensive Care Amsterdam UMC Amsterdam The Netherlands; ^2^ Laboratory of Experimental Intensive Care and Anesthesiology Amsterdam UMC Amsterdam The Netherlands; ^3^ Department of Anesthesiology Amsterdam UMC Amsterdam The Netherlands; ^4^ Department of Pathology Amsterdam UMC Amsterdam The Netherlands; ^5^ Department of Product and Process Development Sanquin Blood Bank – Amsterdam Amsterdam The Netherlands; ^6^ Department of Blood Cell Research Sanquin Research and Landsteiner Laboratory – Amsterdam Amsterdam The Netherlands

**Keywords:** animal, hemodynamics, pulmonary edema, TACO, transfusion reaction

## Abstract

**Background and Objectives:**

Transfusion‐associated circulatory overload (TACO) is the primary cause of transfusion‐related mortality. Speed and volume of transfusion are major risk factors. The aim of this study was to investigate the interaction of red blood cell (RBC) transfusion speed and volume on the development of TACO.

**Materials and Methods:**

A validated model for TACO in anaemic Lewis rats with an acute myocardial infarction was used. The effect on pulmonary hydrostatic pressure of one, two or four units of packed RBCs transfused in either 30 or 60 min was evaluated (3.3–26.6 ml·kg^−1^·hr^−1^). Pulmonary capillary pressure was measured as left ventricular end‐diastolic pressure (LVEDP). Cardiac stress biomarkers atrial natriuretic‐peptide (ANP) and N‐terminal pro‐brain natriuretic peptide (NT‐proBNP) were measured 1‐h post‐transfusion.

**Results:**

Thirty animals were included (*n* = 5 per group). Transfusion of RBCs increased LVEDP in a volume‐dependent manner (ΔLVEDP [mmHg]: −0.95, +0.50, +6.26, *p* < 0.001). Fast transfusion increased overall ΔLVEDP by +3.5 mmHg and up to +11.8 mmHg in the four units' group (*p* = 0.016). Doubling transfusion speed increased ΔLVEDP more than doubling volume in the larger volume groups. No difference in ANP or NT‐proBNP were seen in high transfusion volume or groups.

**Conclusion:**

Transfusion volume dose‐dependently increased LVEDP, with speed of transfusion rapidly elevating LVEDP at higher transfusion volumes. ANP and NT‐proBNP were not impacted by transfusion volume or speed in this model. TACO is seen as purely volume overload, however, this study emphasizes that limiting transfusion speed, as a modifiable risk factor, might aid in preventing TACO.

## INTRODUCTION

Transfusion‐associated circulatory overload (TACO) is the leading cause of transfusion‐related respiratory distress, ICU admission and death [1, 2, 3]. The incidence of TACO in those transfused is 1% of in‐hospital patients [4, 5], and up to 6% in the ICU [6, 7]. Both the speed and volume of transfusion are associated with TACO [5, 6, 8]. Even though millions of transfusions are carried out each year, surprisingly few studies address transfusion speed or volume.

TACO is the result of hydrostatic pulmonary oedema, where transudate is driven into alveoli restricting gas exchange [9, 10]. Both speed of transfusion and large volumes acutely increase circulating volume which can back‐up the pulmonary circulation and increase pulmonary capillary pressure (P_cap_) [11]. Our previously published animal model has shown that after an equivalent volume, blood transfusion increases P_cap_ more than crystalloids [12]. Similarly, in ICU patients transfusion increases P_cap_ and is associated with increased mortality [13]. Only two studies in humans have investigated P_cap_ related to transfusion speed, which increases in a speed‐dependent fashion [14, 15], transfusion volume however has not previously been investigated.

Investigating modifiable risk factors including speed and volume effects of transfusion individually as well as their interaction is necessary to understand TACO. Measuring P_cap_ is difficult and can differ throughout the pulmonary capillary bed, therefore, left‐atrial pressure (LAP ‐ the pressure downstream) is used in clinical research. In humans, this requires a Swan‐Ganz catheter to estimate LAP which is invasive and not without risk [16]. In animals direct intra‐cardiac measurement of left ventricular end‐diastolic pressure (LVEDP), which is equal to the maximum LAP_,_ can be investigated through [12].

The study aimed to investigate the relative contribution of transfusion speed and volume on the development of TACO. Atrial natriuretic peptide (ANP) and N‐terminal pro‐brain natriuretic peptide (NT‐proBNP) will be evaluated as volume overload biomarkers. We hypothesize that both a rapid speed of infusion, as well as larger transfused volumes will increase LVEDP and cardiac stress biomarkers. Using a previously validated rat model of TACO, animals will be randomized and the effect of one, two or four units of packed red blood cells (RBC) transfused in either 30 or 60 min on LVEDP will be measured.

## MATERIALS AND METHODS

This experiment was approved by the Dutch Central Commission for Animal Experiments (licence: AVD118002017814). Experiments were performed in accordance with The Guide for the Care and Use of Laboratory Animals and results were reported according to ARRIVE guidelines. Adult male Lewis rats between 300 and 350 grams were used (LEW/SsNHsd, Envigo, UK) because of reproducibility of myocardial ischemic damage [17]. Animals were housed in a specialized animal care facility, exposed to standard light–dark cycle and fed standard rat chow with ad‐libitum access to water.

**FIGURE 1:**
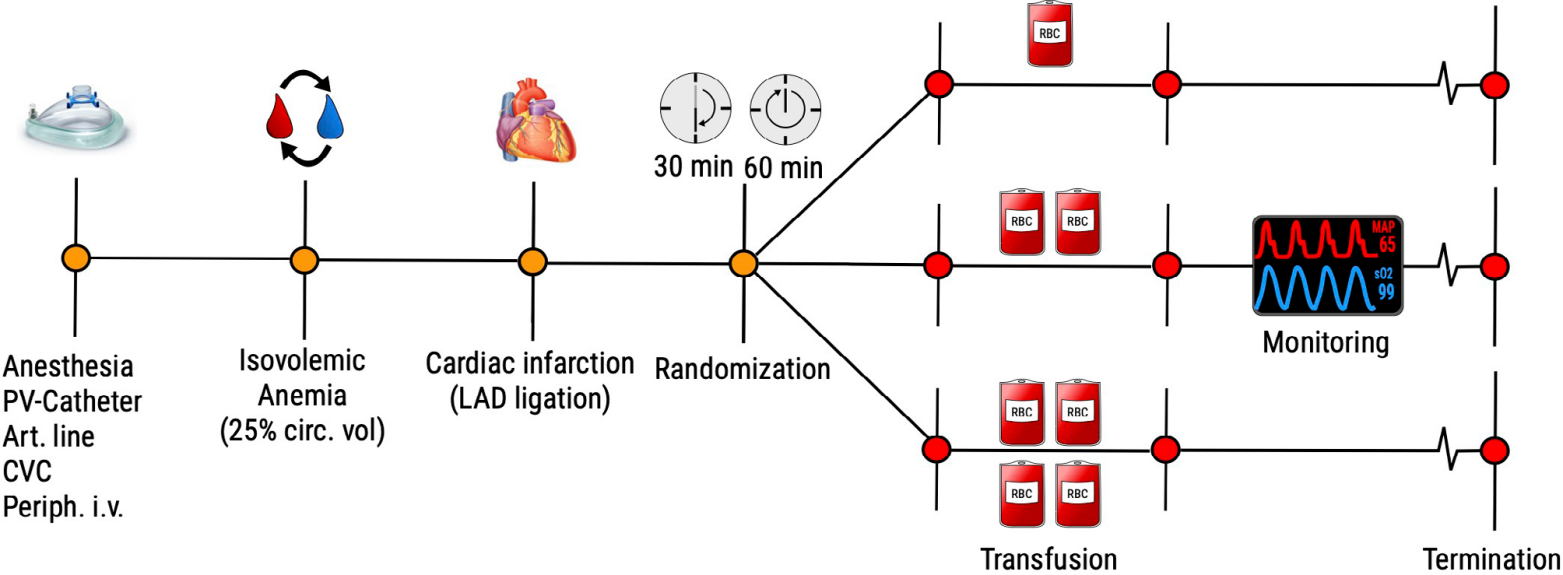
Experiment design comparing speed versus volume of transfusion. A two‐hit model for TACO in anaemic rats was employed. The first hit, an MI results in volume incompliance. Animals were randomized to speed of transfusion (30 or 60 min) and further randomized to receive one, two or four units – a total of six groups. Art.line, Arterial cannula; CVC, Central venous cannula; LAD, Left anterior descending coronary artery; PV‐Catheter, Pressure‐volume (catheter)

A two‐hit model for TACO was used as previously published [12], which is a clinically relevant anaemia‐transfusion model in a mechanically ventilated, cardiac comprised setting (Figure 1). Two hits are required for TACO to develop as healthy anaemic animals were able to accommodate large transfusion volumes. Anaemia was induced by replacing blood with an equivalent volume of colloids, maintaining circulating blood volume (CBV). Isovolemia is important as intravascular hypovolemic patients will be able to accommodate volume, evidenced by the highest incidence of TACO in normovolemic patients with chronic anaemia [18, 19] and INR correction through plasma transfusion in non‐bleeding patients [20]. The first hit is a patient risk factor, resulting in volume‐incompliance, in this case myocardial dysfunction [21]. This lowers the threshold to develop TACO following a second hit – a blood transfusion.

### Animal procedures

General anaesthesia was induced in healthy animals through brief use of isoflurane (5%) followed by an intra‐peritoneal injection of racemic ketamine (9 mg·100 g^−1^), dexmedetomidine (12.5 μg·100 g^−1^) and atropine (5 μg·100 g^−1^). Ketamine was continuously infused through a tail‐vein cannula to maintain anaesthesia (5 mg·100 g^−1^·h^−1^), chosen for its limited cardiovascular depressive profile. Animals were ventilated via a tracheostomy secured with a suture around the trachea.

#### Cardiac catheterization and line placement

An ultraminiature rat pressure‐volume catheter (SPR‐838, Millar, USA) was passed through the right carotid artery into the left‐ventricle. Calibrations of the PV‐catheter include: a two‐point pressure calibration, blood conductivity calibration using the cuvette method and correction for parallel conductance using hypertonic saline boluses (NaCl 30%, 10 μl bolus) [22]. Conductivity calibrations were performed at fixed timepoints following changes in blood electrolyte, that is: (1) baseline, (2) pre‐transfusion, (3) post‐transfusion and (4) at termination. The right jugular vein was cannulated to record central venous pressure (CVP) and the left carotid artery to measure mean arterial pressure (MAP) and perform blood draws.

#### Isovolemic anaemia and first hit – Myocardial ischemia

Approximately 20% of CBV was replaced over a period of 15 min with an equivalent volume of colloid solution (Tetraspan 6%, B. Braun, Germany) until a haematocrit of 30% was achieved. CBV (ml) was calculated as 6.5% of body weight (g) [23]. Afterwards animals were allowed to stabilize for 15 min.

A myocardial infarction (MI) was induced as first hit. A continuous norepinephrine infusion was started and titrated to a MAP of 65 mmHg. Through a left‐anterior thoracotomy (±2 cm incision through the fourth intercostal rib) the left‐anterior descending artery (LAD) was permanently ligated using a 5‐0 Prolene suture [12, 24]. Ischemia was confirmed visually by blanching of the myocardium distal to the ligation and ST‐elevations on a three‐lead ECG. An intrapleural chest drain (20G) was placed one rib below the incision and the thorax and skin were closed in two layers using a 3‐0 Vicryl suture. After closing the chest, the drain was removed under negative pressure while simultaneously a recruitment manoeuvre was performed to minimize residual air. Animals were allowed to stabilize for 30 min.

#### Randomization and second hit – Transfusion

A sealed envelope, block randomization method was used to stratify animals to either one, two, or four units to be transfused using a volumetric pump, performed in equal groups per week (a 1:1:1 ratio). Animals were then again randomized by sealed envelope to rapid or slow transfusion (over 30 or 60 min). From randomization onward, no further alterations were allowed to ventilator settings, fluid, norepinephrine or anaesthetic infusion rates. Volume of RBC units was predetermined and fixed (1.0 ml per unit) calculated as 5% of CBV – equivalent to 80 kg male humans. Hemodynamic parameters were recorded continuously until the experiment ended at 1‐h post‐transfusion and the rats were terminated. The RBC units were buffy coat reduced packed red blood cells products, manufactured following Dutch blood banking procedures, made from the whole blood harvested from donor animals outside the experimental group (Supplementary Appendix A).

#### Tissue harvesting and laboratory methods

Animals were exsanguinated 1‐h post‐transfusion. Collected blood was processed within 2 h, centrifuged at 2000*g* for 10 min and the supernatant stored at −80.0°C for later analysis. Rat ANP and NT‐proBNP levels were determined according to manufactures guidelines (products: E‐EL‐R0670/R0017, Elabscience, China). The lungs were harvested, the right upper lobe was fixed in formalin for histopathological analysis and the lower lobe used to calculate the wet‐dry ratio: wet‐weight/dry‐weight. The myocardial infarct size (volume percentage) was determined to ensure equal infarct sizes between groups (Supplementary Appendix B).

#### Outcomes

The primary outcome was ∆LVEDP (post minus pre‐transfusion LVEDP) compared between all six groups that is, number of units transfused (one, two or four units) and speed of transfusion (rapid or slow). Secondary outcomes included change in heart rate (HR), mean arterial pressure (MAP), cardiac output (CO), central venous pressure (CVP), systemic vascular resistance (SVR), stroke work (calculated as the area within the PV‐loop) and cardiac relaxation (−*dP*/*dt*). Biomarkers included the difference in ANP and NT‐proBNP between groups. Pulmonary outcomes included oxygenation capacity PaO_2_/FiO_2_‐ratio (PF‐ratio), wet‐dry ratio and histopathological examination.

#### Sample size

Sample size was determined using previously published data [12], and further extrapolated assuming a linear correlation between ∆LVEDP and the number of units transfused. The median ∆LVEDP following four units of RBCs was +8.0 mmHg (IQR 7.5–14.1). Based on an α of 0.05 and β of 20% we calculated that *n* = 3 animals in each arm were required to detect a clinically relevant difference [18] (4.0 mmHg difference in ∆LVEDP) between one and four units transfused. A total of five animals per group was chosen to account for variation in the model.

#### Statistical analysis

Statistical analysis was performed in R Statistics v3.3.2 (R Foundation for Statistical Computing, Austria), using RStudio v1.0.136 (RStudio, Inc, USA). Data were assessed for normality using histograms. Cardiac parameters including ΔLVEDP (post‐transfusion minus pre‐transfusion) and biomarkers were compared between the number of units transfused using the Kruskal‐Wallis test and post‐hoc Mann–Whitney‐*U* test. Effects dependent on speed of transfusion were compared using the Mann–Whitney‐*U* test. Lung pathology scores were compared using a chi‐squared test. A *p*‐value of <0.05 was considered significant.

## RESULTS

A total of 30 animals were included, randomized to one, two or four units transfused (*n* = 10 per group) and further randomized to either slow or rapid infusion (*n* = 5 per group). Prior to randomization seven animals died during cannulation due to cardiac tamponade (*n* = 2), during thoracotomy (*n* = 1) or due to arrhythmia's following myocardial infarction (*n* = 4). Animals that died prior to randomization were replaced; no animals died after randomization.

### Isovolemic anaemia, validation of myocardial dysfunction and characteristics

Isovolemic anaemia resulted in a significant haematocrit decreased from median 43% (IQR: 42–44) to 30% (IQR: 29–31, *p* = 1.70 × 10^−6^). The effects of the first hit were seen as (1) a ventricle infarction percentage of 43% (IQR: 38–49); (2) stroke work decreased from baseline by −1347 mmHg∙μl (IQR: 847–1781, *p* = 4.90 × 10^−4^); and (3) −*dP*/*dt*, a parameter for left‐ventricular relaxation, worsened from baseline −6874 mmHg·s^−1^ (IQR: −7274 to −6404) to −5244 (IQR: −5587 to −4586, *p* = 1.60 × 10^−7^). Because of left‐ventricular conformational changes, following ligation of the LAD, ejection fraction has proven to be unreliable [12]. Characteristics at the time of randomization are shown per number of units transfused in Table 1. Following transfusion haematocrit increased significantly for all groups, from 29.7 ± 1.6 prior to transfusion, to 39.7 ± 1.8 (*p* = 2.02 × 10^−7^) for one unit, 43.8 ± 1.6 (*p* = 2.02 × 10^−9^) for two and 48 ± 2.4 (*p* = 2.76 × 10^−9^) for four units.

**TABLE 1:** Characteristics pre‐transfusion

Characteristics	1 unit (*n* = 10)		2 units (*n* = 10)		4 units (*n* = 10)	
	Slow (*n* = 5)	Fast (*n* = 5)	Slow (*n* = 5)	Fast (*n* = 5)	Slow (*n* = 5)	Fast (*n* = 5)
Weight (g):	335 (325–340)	325 (315–340)	325 (320–325)	320 (310–330)	315 (315–335)	330 (315–330)
P/F‐ratio:	343 (247–381)	394 (366–423)	423 (395–453)	366 (333–396)	370 (281–440)	374 (374–408)
LVEDP (mmHg):	10.9 (9.9–14.3)	10.3 (9.8–12.3)	12.9 (11.2–14.1)	10.6 (9.5–10.9)	10.4 (9.3–13.0)	9.5 (7.5–11.9)
Heart rate (min^−1^):	253 (221–257)	204 (193–225)	245 (236–250)	238 (231–263)	287 (257–289)	244 (239–246)
Mean arterial pressure (mmHg):	65 ± 7	72 ± 8	63 ± 8	63 ± 12	62 ± 13	59 ± 8
LVP_max_ (mmHg):	98 (90–103)	102 (95–103)	99 (93–102)	90 (85–99)	100 (93–102)	115 (95–117)
Stroke volume (μl):	52 (46–57)	55 (37–70)	58 (57–60)	59 (55–62)	46 (34–59)	70 (63–75)
Cardiac output (ml·min^−1^)	13 (12–15)	11 (7–17)	14 (14–16)	15 (13–15)	13 (9–15)	17 (15–18)
Ejection fraction (%):	65 (38–67)	50 (33–59)	44 (35–58)	47 (44–49)	66 (51–80)	48 (47–53)
Myocardial infarct (%):	47 (43–52)	44 (43–48)	46 (45–56)	41 (40–43)	39 (34–46)	37 (33–50)
Rate pressure product (mmHg·min^−3^·10^3^):	22.9 (22.8–23.0)	20.7 (20.7–20.8)	22.9 (22.8–26.4)	21.2 (19.8–22.8)	29.6 (24.0–30.6)	26.9 (23.3–28.0)
Stroke work (mmHg·μl·10^3^):	3.9 (3.9–4.5)	4.0 (3.8–4.8)	4.5 (4.1–4.8)	5.0 (4.2–5.4)	4.2 (3.5–4.7)	5.5 (5.1–6.4)
Central venous pressure (mmHg):	5.3 ± 1.15	3.9 ± 0.93	6.3 ± 2.97	6.5 ± 2.24	5.3 ± 1.71	4.5 ± 0.89
Systemic vascular resistance (dyn·s·cm^−5^):	373 (310–377)	381 (303–524)	317 (303–338)	415 (339–417)	332 (259–401)	235 (230–300)
Norepinephrine (μg·kg^−1^):	6.5 (3.6–11.4)	7.7 (6.2–9.5)	5.0 (4.0–7.9)	7.7 (3.9–9.1)	8.8 (2.5–12.9)	12.1 (2.5–13.3)
Fluid balance (ml):	2.6 (2.5–2.7)	2.3 (2.1–2.6)	2.5 (2.4–2.5)	2.7 (2.5–2.8)	2.3 (2.2–2.9)	3.3 (3.1–4.7)

*Note*: Characteristics at randomization, prior to transfusion. Results presented as median (IQR) or mean ± SD.

Abbreviations: LVEDP, left ventricular end‐diastolic pressure; LVP_max_, left‐ventricular maximum pressure; P/F‐ratio, PaO_2_/FiO_2_‐ratio.

### Changes in LVEDP between volume transfused and speed of infusion

**FIGURE 2:**
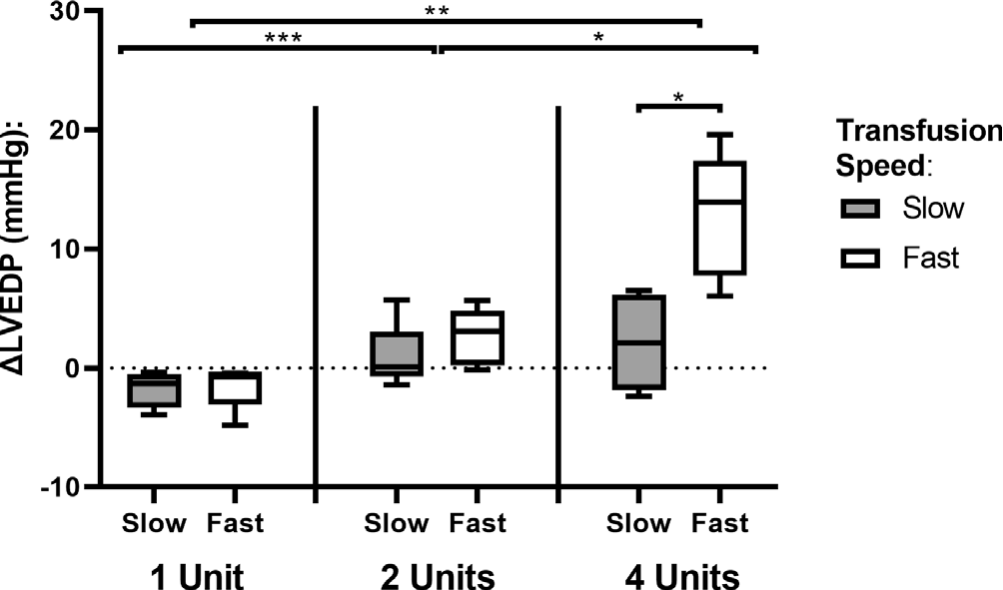
Change in LVEDP per transfusion volume and speed. Data presented in a Tukey boxplot. ∆LVEDP increases significantly with more units transfused (top brackets). Speed was significantly associated with an increase in ∆LVEDP only in the group transfused with four units. *: *p* < 0.05; **: *p* < 0.01; ***: *p* < 0.001

Depending on the number of units transfused the overall ∆LVEDP increased significantly (Kruskal‐Wallis test, *p* = 8.06 × 10^−4^), with ∆LVEDP after one unit −0.95 (IQR: −2.4 to −0.5), two units 0.50 (IQR: 0.0–3.7) and four units 6.3 (IQR: 3.0–12.8). Post‐hoc testing showed an increase between one versus two units (*p* = 4.87 × 10^−4^), two versus four units (*p* = 0.043) and one versus four units (*p* = 0.003). Speed was significantly associated with an increase in ∆LVEDP only in the group transfused with four units (Figure 2 *)*.

A secondary analysis was performed to determine the contribution of speed compared to transfused volume on LVEDP. Both doubling of speed and volume of transfusion increased ∆LVEDP (Table 2). At low volumes the changes were not‐clinically relevant, however at larger volumes, a doubling of transfusion speed resulted in an exponential increase ∆LVEDP (four‐times higher).

**TABLE 2:** Effect of speed compared to volume on LVEDP

	Doubling infusion speed			Doubling infusion volume		
	Fast infusion ∆LVEDP (mmHg)	CI 95%	*p*‐value	Volume ∆LVEDP (mmHg)	CI 95%	*p*‐value
1 Unit ‐ Slow	+0.74	−3.52 to 3.41	0.841	+1.37	−0.11 to 6.98	0.056
2 Units ‐ Slow	+3.01	−2.60 to 5.59	0.421	+2.05	−6.52 to 3.56	0.691
4 Units ‐ Slow	+11.77	2.94 to 17.60	0.016	‐	‐	‐

*Note*: Side‐by‐side comparison of effects of doubling transfusion speed versus doubling transfused volume on ∆LVEDP. Results presented as median (CI 95%).

### Changes in haemodynamics following transfusion

With increasing transfused volume and increased preload, seen as increased LVEDP, no increase in overall CO was detected (Table S1, *p* = 0.468). In addition, stroke work decreased in all groups (*p* = 2.48 × 10^−3^). Also, overall HR (+21.7 bpm [IQR: 20.2–27.7, *p* = 3.90 × 10^−7^]), MAP (+15.4 mmHg [IQR: 10.3–22.1, *p* = 4.40 × 10^−5^]) and SVR increased significantly (+97.7 dyn·s·cm^−5^ [IQR: 77.1–167.1, *p* = 2.48 × 10^−3^]).

### Pulmonary outcomes

Overall, no differences were found in pulmonary outcomes (Table S2). P/F‐ratio at termination nor wet‐dry ratio or histopathological examination of the lungs (where pulmonary oedema was ranked on a 0–3‐point scale by an experienced pathologist) differed between the number of units transfused, nor the speed of transfusion.

### Biomarkers

**FIGURE 3:**
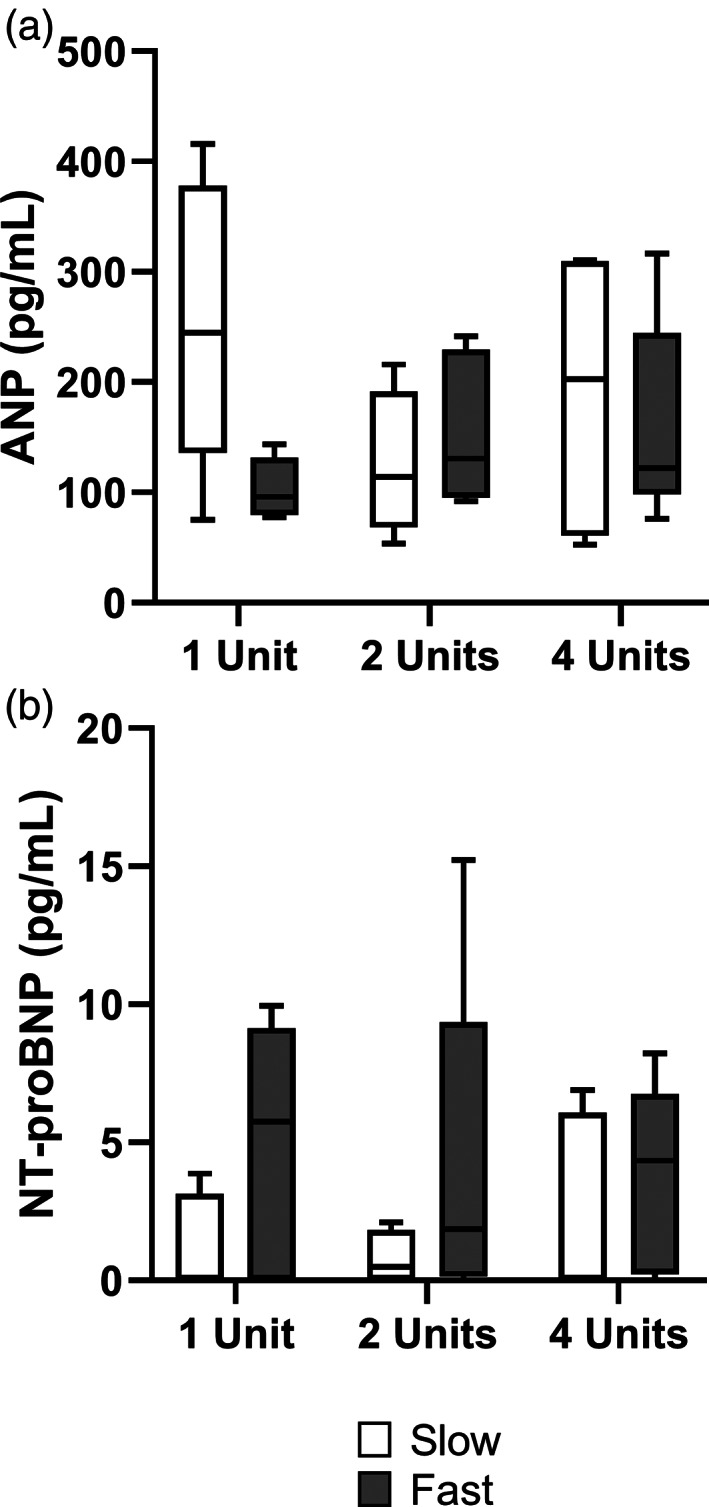
Volume overload biomarkers. Data presented in a Tukey boxplot

Overall, ANP and NT‐proBNP were elevated (Figure 3), however they were not significantly difference in between the number of units (respectively *p* = 0.894 and *p* = 0.931) or the speed of transfusion (respectively *p* = 0.931 and *p* = 0.074). Secondary analyses including log‐transformation did not change results.

## DISCUSSION

TACO can result in major morbidity including ICU admission, mechanical ventilation and death. Both speed and volume of transfusion are modifiable risk factors for developing TACO [8, 25]. The primary findings of this study are (1) the increase in hydrostatic pulmonary capillary pressure is transfusion speed and volume‐dependent; (2) increasing transfusion speed or volume both increase LVEDP; and (3) both ANP and NT‐proBNP biomarkers of cardiac overload were not significantly increased in this animal model within 1‐h following transfusion.

An increased LAP is the mechanism behind developing TACO. A landmark study in dogs showed that hydrostatic pulmonary oedema started developing at pressures above 25 mmHg [26]. There is limited evidence of how speed and volume individually contribute to the development of TACO. Mechanistic transfusion studies have been performed in both healthy dogs [27] and swine [28]. Both show an increase in pulmonary pressures following transfusion, similar to our results. Two studies in humans [15, 18] showed a transfusion speed‐dependent increase, also similar to our model. Our model is the first to employ packed red blood cells contrary to use of whole blood. Rheological properties of units with 60% haematocrit are likely to alter hemodynamic outcomes unpredictably. Second, this is the first study to investigate both transfused volume and speed. There is a clear stepwise increase in LVEDP as result of increasing transfusion volume. Transfusion speed seems particularly important to higher transfusion volumes doubling of speed increased LVEDP exponentially (Figure 1 and Table 2). Our data suggesting transfusion speed is a critical factor is an important signal to clinicians that could prevent TACO.

Successful interventions to reduce over‐transfusion include patient blood management programs, transfusion triggers and a single‐unit transfusion in non‐emergency situations [29]. Splitting an RBC unit can also reduce the volume infused over time, allowing transfusion over days without exposing a patient to multiple donors. Finally, volume‐reduced RBC units, hyperconcentrated platelets and partially reconstituted lyophilized plasma have been suggested to reduce the volume load of transfusion [9, 30]. Reducing transfusion speed in non‐emergency settings is an easy intervention, even so, there is likely room for improvement as retrospectively in 50% of TACO cases infusion speed was not specified [31]. There is limited guidance on speed of transfusion, aside from a 4‐h maximum to prevent bacterial growth. Noted is that transfusion speed may be higher if tolerated by the patients circulatory system [32]. Specifically, in at‐risk patients guidance regarding the speed of transfusion is warranted.

### Cardiac overload biomarkers

While markers are elevated, ANP and NT‐proBNP levels did not contribute in discerning different volumes or speeds of transfusion (Figure 3). ANP is used experimentally and is secreted from preformed vesicles and the atria, ready for rapid release [33]. NT‐proBNP is widely used in clinical practice as an intermediate to long‐term biomarker (hours to days) of congestive heart failure, rising over hours. Elevated levels of biomarkers are not required for the diagnosis of TACO, but rather provide additional evidence of circulatory overload. The lack of a volume‐dependent increase in these biomarkers may be explained by the follow‐up of only 1‐h. While transfusion directly increases preload, the negative spiral of progressive heart failure and overdistention might require up to 12 h to manifest in line with TACO criteria [10]. Also, the myocardial damage following an MI may confound biomarker results in this model. Further study is required to assess the value of biomarkers in TACO.

An inherent limitation to our model is the fluid balance in rats, as they consume daily a volume of water equal to their CBV. Since rats are used to large changes in volume per day an RBC unit is less likely to overwhelm the circulation, where one unit was calculated as 5% of CBV like humans. Furthermore, we chose a limited follow‐up duration of 1 h, as not to induce bias. Previous experiments have shown that it is difficult to keep animals stable for longer than 1 h without interventions. Fluid boluses, vasopressor or ventilatory changes introduce bias and are difficult to correct for. Another limitation is baseline differences at randomization (Table 1), showing a higher fluid balance and norepinephrine dose in the group receiving 4‐units fast. Based on both CVP and LVEDP this group does not appear to have an increased preload thereby directly increasing the risk of TACO. Increased norepinephrine after cardiac surgery hints at a systemic inflammatory response, the degree of which will always differ between subjects. Finally, we did not find a transfusion volume or speed‐dependent increase in pulmonary oedema. Rather this is a hydrostatic pressure model, an advantage being the use of syngeneic rats, which limits the immunological component of transfusion. It cannot be ruled out that pulmonary oedema could still develop within the 12‐h TACO window. The results of this model remain relevant, as even in the absence of pulmonary oedema transfusion in ICU patients is associated with both an increased LAP as well as increased mortality [13].

We utilized a specialized rat model involving an acute MI and transfusion. This model provides a mechanistic approach to circulatory overload and simplifies how TACO develops in humans, who are often older and have different predisposing conditions. We have previously shown that transfusion increases LVEDP not only after an MI but also with underlying acute kidney injury (AKI) [14]. Therefore transfusion of RBC's increases P_cap_ in volume incompliant rats independent of cardiac function, which broadens the generalizability of our results using an MI model.

Interestingly a recent RCT found very low incidences of acute heart failure following liberal transfusion (3.7%) versus restrictive transfusion (3.2%, n.s.) [34]. Unfortunately, the study did not specifically score TACO, nor was the speed of transfusion documented. The low incidence of TACO may additionally be explained by other interventions such as routine echocardiograms and two‐thirds of patients were either already using or started on diuretics within 24‐h of admission, all of which being interesting targets for TACO research.

Translating our findings will inevitably require humans studies to guide transfusion speed. While slower is likely better, not all patients will require this, and an individual risk‐assessment should be performed. Future studies using this model should also focus on interventions to prevent and treat TACO, for example through loop‐diuretics or volume‐reduced blood products. The upcoming challenge will be the development of a TACO phenotype model with evident pulmonary oedema, in which interventions can start targeting pulmonary oedema. Future studies in humans should make sure transfusion speed is equal between groups. This study paves the way to a study in high‐risk patients (i.e., non‐bleeding patients >60‐years‐of‐age with either pre‐existing renal or cardiac dysfunction) to be randomized to receive either a split or full RBC unit over 1 or 2 h.

In conclusion, this study demonstrates a stepwise transfusion volume‐dependent increase of LVEDP which is key in the development of TACO. Transfusion speed appears critical in developing circulatory overload, specifically when larger volumes were transfused. Cardiac biomarkers ANP and NT‐proBNP were not correlated with the volume or transfusion speed 1‐h after transfusion. This study underlines a policy of slow transfusion in non‐urgent cases.

## CONFLICT OF INTEREST

The authors declare that they have no competing interest.

## Supporting information


**Data S1.** Supporting information.

**Data S2.** Tables.
