# Supplementary material for: Differential effects of speed and volume on transfusion‐associated circulatory overload: A randomized study in rats
Source: Vox Sang. 2021 Aug 15;117(3):371–8. doi: 10.1111/vox.13191 (PMC9291097; doi:10.1111/vox.13191)
Supplement: Supplementary file 1 — Data S1. Supporting information. [file VOX-117-371-s001.docx]

**Supplementary appendix A.**

RBC units were buffy coat reduced products, made from the whole blood harvested from donor animals.[1] Donor animals were anesthetized using isoflurane 5% and exsanguinated through a closed-chest left-ventricular puncture. Whole blood was collected in a citrate-phosphate-dextrose solution and pooled from multiple donors. In-bred rats are syngeneic and have no blood type, resembling an autologous transfusion and limiting allogeneic inflammatory reactions associated with transfusion-related acute lung injury. After centrifugation (10 minutes at 2000g), the plasma and buffy coat were removed, and the red cell pellet resuspended in a saline-adenine-glucose and mannitol solution, to a target hematocrit of 60%. All transfused animals during that week received blood from the same batch, limiting inter-product variability, the pooled product was used within five days of preparation.

**Supplementary appendix B.**

The heart was harvested with the LAD suture in place and myocardial tissue was stained to quantify cardiac infarct sizes ensuring equal infarct sizes between groups.[1] The aorta was cannulated and the coronary circulation anterogradely perfused with 1) 0.9% sodium-chloride heparin solution 1.0 IE·mL^-1^ to prevent thrombus formation; 2) Evans Blue dye (2%) for three minutes to discriminate viable from infarcted cardiac tissue distal to the ligation 3) excess dye was flushed with the sodium-chloride heparin solution. Thereafter, samples were frozen at -20.0^∘^C to be cut into 2mm transverse slices and counterstained using triphenyltetrazolium chloride (7.5 mg·mL^-1^) for 15 minutes at 37°C with subsequent fixing of the tissue in 4% formalin. Slices were scanned at high-resolution, and the area infarcted tissue was calculated using ImageJ v1.52 (National Institute of Health – *USA*) and the volume calculated based on the slice thickness.

**REFERENCES**

1. Klanderman R, Bosboom J, Maas M, et al. Volume incompliance and transfusion are essential for Transfusion-Associated Circulatory Overload: a novel animal model. *Transfusion* 2019;59:3617–27.
