# Supplementary material for: Differential effects of speed and volume on transfusion‐associated circulatory overload: A randomized study in rats
Source: Vox Sang. 2021 Aug 15;117(3):371–8. doi: 10.1111/vox.13191 (PMC9291097; doi:10.1111/vox.13191)
Supplement: Supplementary file 2 — Data S2. Tables. [file VOX-117-371-s002.docx]

**Table S1.** Change in hemodynamic parameters after transfusion

| **Change in  hemodynamics** | **1 Unit (n = 10)** | | **2 Units (n = 10)** | | **4 Units (n = 10)** | | **Units transfused* (p-value)** | **Speed of transfusion** (p-value)** |
| --- | --- | --- | --- | --- | --- | --- | --- | --- |
|  | **Slow (n=5)** | **Fast (n=5)** | **Slow (n=5)** | **Fast (n=5)** | **Slow (n=5)** | **Fast (n=5)** |  |  |
| ∆LVEDP (mmHg): | -1.3 (-2.8 – -0.6) | -0.6 (-1.3 – -0.5) | 0.1 (-0.3 – 0.4) | 3.1 (0.6 – 3.9) | 2.1 (-1.3 – 5.8) | 13.9 (9.4 – 15.2) | <0.001 | 0.002 |
| ∆Heart rate (min^-1^): | 22  (21 – 24) | 23 (20 – 33) | 21 (15 – 32) | 7 (-1.4 – 13) | 39 (32 – 42) | 17 (7 – 23) | 0.342 | 0.137 |
| ∆MAP (mmHg): | 0 (-1 – 10) | 2 (-3 – 17) | 22 (9 – 34) | 18 (2 – 30) | 29 (13 – 37) | 37 (19 – 51) | 0.010 | 0.775 |
| ∆LVP_max_ (mmHg): | 4 (0 – 15) | -4 (-6 – 15) | 20 (17 – 24) | 19 (3 – 26) | 26 (19 – 35) | 10 (10 – 25) | 0.029 | 0.267 |
| ∆SV (μL): | -9.8 (-10 – -7.4) | -4.8 (-13 – -2.3) | -7.8 (-11 – -4.0) | 8.2 (1.4 – 13) | -17 (-18 – 8.3) | -17 (-19 – -9.5) | 0.132 | 0.402 |
| ∆CO (mL·min^-1^): | -1 (-2 – 0) | 0 (-1 – 0) | -1 (-1 – 0) | 3 (2 – 3) | 0 (-4 – 2) | -3 (-4 – -1.5) | 0.468 | 0.488 |
| ∆RPP (mmHg·min^-3^·10^3^): | 3.2 (2.4 – 4.2) | 1.8 (0.3 – 7.1) | 8.6 (6.5 – 9.8) | 4.4 (0.6 – 8.5) | 10 (9.5 – 11) | 5.4 (4.8 – 7.0) | 0.124 | 0.098 |
| ∆Stroke work (mmHg·μL·10^3^): | -1.3 (-1.7 – -0.6) | -0.6 (-0.9 – 0) | -0.5 (-0.8 – -0.3) | -0.6 (-1.6 – 0.3) | -1.3  (-1.8 – -0.5) | -2.1 (-2.4 – -1.9) | 0.082 | 0.687 |
| ∆CVP (mmHg): | 0.2 (-0.1 – 0.3) | -0.8 (-1.4 – -0.2) | 0.1 (0.1 – 0.2) | -1.2 (-4.1 – 1.3) | -0.9 (-1.5 – -0.3) | 0.7 (-0.5 – 0.9) | 0.870 | 0.861 |
| ∆SVR (dyn·s·cm^-5^): | 17 (13 – 117) | 41 (22 – 56) | 100 (34 – 195) | 0 (-63 – 55) | 73 (60 – 153) | 187 (112 – 258) | 0.296 | 0.584 |

Results presented as median (IQR). *Comparing one-, two- and four-unit groups. **Comparing rapid versus slow transfusion speed. *Abbreviations*: *LVEDP*: left-ventricular end diastolic pressure; *MAP*: mean arterial pressure; *LVP_max_*: left-ventricular maximum pressure; *SV*: stroke volume; *CO*: cardiac output; *RPP*: rate pressure product; *CVP*: central venous pressure; *SVR*: systemic vascular resistance.

**Table S2.** Pulmonary outcomes

| **Change in  hemodynamics** | **1 Unit (n = 10)** | | **2 Units (n = 10)** | | **4 Units (n = 10)** | | **Units transfused* (p-value)** | **Speed of transfusion** (p-value)** |
| --- | --- | --- | --- | --- | --- | --- | --- | --- |
|  | **Slow (n=5)** | **Fast (n=5)** | **Slow (n=5)** | **Fast (n=5)** | **Slow (n=5)** | **Fast (n=5)** |  |  |
| P/F-ratio at termination: | 422 (381–463) | 403 (377–440) | 413 (376–454) | 396 (234–480) | 397 (281–436) | 373 (265–416) | 0.940 | 0.801 |
| Wet-dry ratio: | 4.7 (4.5–4.8) | 4.7 (4.6–4.8) | 5.0 (4.9–6.0) | 5.0 (4.7–5.0) | 4.7 (4.6–4.8) | 4.8 (4.6–5.0) | 0.382 | 0.621 |
| Histopathological score: | 2 (2–2) | 2 (2–2) | 1 (1–2) | 1 (1–2) | 2 (1–2) | 2 (2–3) | 0.247^†^ | 0.190^†^ |

Results presented as median (IQR). *Comparing one-, two- and four-unit groups. **Comparing rapid versus slow transfusion speed. † Chi-square analysis. *Abbreviations*: *P/F-ratio*: PaO_2_/FiO_2_-ratio.
